# Opposed expression of IKKα: loss in keratinizing carcinomas and gain in non-keratinizing carcinomas

**DOI:** 10.18632/oncotarget.4548

**Published:** 2015-08-06

**Authors:** Desheng Xiao, Jiantao Jia, Ying Shi, Chunyan Fu, Ling Chen, Yiqun Jiang, Li Zhou, Shuang Liu, Yongguang Tao

**Affiliations:** ^1^ Department of Pathology, Xiangya Hospital, Central South University, Changsha, Hunan, 410078, China; ^2^ Cancer Research Institute, Central South University, Changsha, Hunan, 410078, China; ^3^ Key Laboratory of Carcinogenesis and Cancer Invasion, Ministry of Education, Hunan, 410078, China; ^4^ Key Laboratory of Carcinogenesis, National Health and Family Planning Commission, Hunan, 410078, China; ^5^ Center for Medicine Research, Xiangya Hospital, Central South University, Changsha, Hunan, 410008, China

**Keywords:** IKKα, keratinizing cancer, non-keratinizing cancer

## Abstract

The functional role of IKKα *in vivo* is pretty complicated, largely due to its diverse functions through cell autonomous and non-autonomous manners. In addition, most of the studies on IKKα were derived from animal models, whether these findings hold true in human tumors remain unclear. Here we examined the expression of IKKα in nasopharyngeal carcinoma, which includes non-keratinizing carcinoma and keratinizing squamous cell carcinoma, and lung squamous cell carcinoma with keratinization and non-keratinization. We demonstrated that IKKα expression was almost negative in keratinizing cancer and higher expression of IKKα was found in non-keratinizing cancer, and that IKKα expression correlateed with cellular differentiation of tumors in non-keratinizing nasopharyngeal carcinoma. These findings demonstrate that IKKα is diversely expressed in keratinizing and non-keratinizing carcinomas in the same type of cancer.

## INTRODUCTION

Nuclear factor (NF)-κB activation leads to a protumorigenic inflammatory microenvironment of various tumors [1]. The NF-κB pathway is tightly stimulated by the IκB-kinase (IKK) complex, which consists of two catalytic subunits, IKKα and IKKβ, and a regulatory subunit, IKKγ [2]. Whereas, in most malignancies, the classical IKKβ/IKKγ-dependent NF-κB activation controls key functions for tumor initiation, promotion and progression in tumors [3].

The noncanonical NF-κB pathway is correlated with IKKα, the role of IKKα in noncanonical NF-κB pathway is more complex [4, 5]. Depending on the type of malignancy, IKKα can provide both tumor-promoting and tumor-suppressive mechanisms that are in most instances cell autonomous. Inhibition of IKKα prolongs survival and suppresses occurrence of metastatic diseases in models of mammary, prostate cancer and colorectal cancer [6–10]. IKKα controls expression of the inhibitor of metastasis maspin in breast and prostate cancer [7, 8] and is required for ErbB2-induced mammary tumorigenesis. In the latter case, NIK-dependent IKKα activation promotes expansion of tumor-initiating cells by directly phosphorylating the cyclin-dependent kinase inhibitor p27 [9].

In contrast, IKKα acts as a tumor suppressor in models of skin or lung squamous cell carcinoma (SCC), loss of IKKα enhances susceptibility to carcinogen-induced SCC in the skin and leads to the development of spontaneous lung SCC in mice [11, 12]. Importantly, during development of lung SCC, IKKα kinase inactivation culminates in the recruitment of tumor-promoting inflammatory macrophages and depletion of macrophages prevents SCC formation [12]. This is in clear contrast to the findings that IKKα promotes intestinal tumorigenesis by limiting recruitment of M1-like polarized myeloid cells [10], yet the reason for this diverse role of IKKα in macrophage activation profile in these two different tumor entities remains currently unclear. However, it is possible that spontaneous lung SCC initiated by inactivation of IKKα belongs to keratinizing carcinoma according to the results [12]. Therefore, we assume that the expression level of IKKα might involve in the distinct subtype of in keratinizing and non-keratinizing carcinomas.

## RESULTS AND DISCUSSION

**Figure 1:**
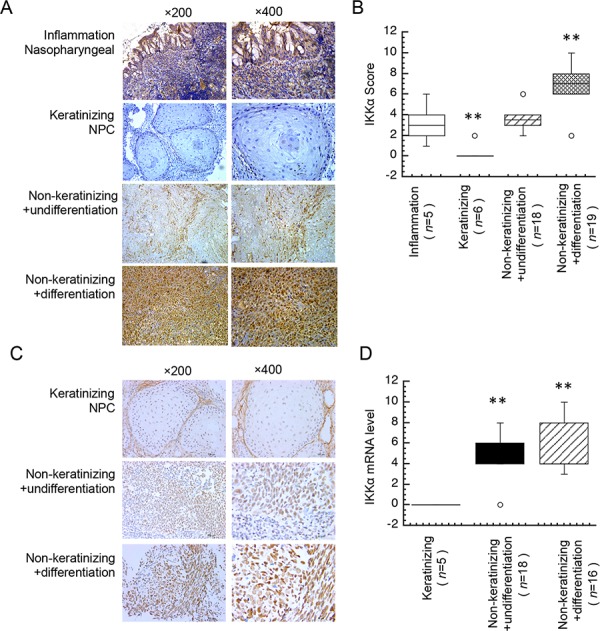
Different expression level of IKKα in NPC tissues **A.** Immunohistochemical analysis was used to examine the protein level of IKKα in an inflamed nasopharyngeal and tissues from NPC patients. **B.** Expression protein level of IKKα in inflamed nasopharyngeal and subtypes of NPC tissues as indicated. **C.** The mRNA level of IKKα was analyzed by ISH in NPC tissues. **D.** Expression mRNA level of IKKα in subtypes of NPC tissues as indicated. *n*, number of analyzed samples, ** *p* < 0.01.

To address the hypothesis that IKKα might involve in the distinct subtype of in keratinizing and non-keratinizing cancer, we used a subtype cancer that contains keratinizing and non-keratinizing cancers. Nasopharyngeal carcinoma (NPC), a prevalent cancer in southern China, is a common epithelial carcinoma in that is related with Epstein-Barr virus (EBV). NPC is classified into two major histological subtypes: non-keratinizing carcinoma (either differentiated or undifferentiated) and keratinizing squamous cell carcinoma [13, 14]. Poor differentiation is a hallmark of solid tumors and correlates with loss of control in tumor growth and poor prognosis of patients. Here we used immunohistochemistry analysis with IKKα primary antibody to detect the expression level of IKKα in three subtypes of the primary human NPC biopsies (keratinizing NPC, non-keratinizing with undifferentiated NPC and non-keratinizing with differentiated NPC). A total of five non-cancer nasopharyngeal and 43 NPC samples were evaluated. High IKKα staining was displayed in differentiated NPC specimens (77.78%), but only in a minority (22.22%) of undifferentiated NPC samples. As expected, weak IKKα staining was displayed in non-keratinizing NPC samples. Data in Figure 1A showed that IKKα was significantly lower in non-keratinizing NPC compared with the normal nasopharyngeal epithelium, while IKKα increased in non-keratinizing NPC and the highest of IKKα was expressed in differentiated NPC. Furthermore, the median score of IKKα expression in undifferentiated NPC specimens was markedly lower compared with the differentiated NPC, moreover, the score of IKKα expression in keratinizing NPC was significantly lower as compared with non-keratinizing NPC and non-cancer tissues (Figure 1B).

Next, to further confirm IKKα level in NPC biopsies, we used *in situ* hybridization (ISH) to analyze the mRNA level of IKKα. A total of 39 NPC samples were evaluated with ISH. Data in Figure 1C showed that IKKα was significantly absence in non-keratinizing, while IKKα increased in non-keratinizing NPC and the highest of IKKα was expressed in differentiated NPC. The median score of IKKα expression in undifferentiated NPC specimens was slightly lower compared with the differentiated NPC, moreover, the score of IKKα expression in keratinizing NPC was significantly lower as compared with non-keratinizing NPC (Figure 1D). Our findings are consistent with recent findings show that IKKα is highly expressed in differentiated NPC and functions as an inhibitor of tumor growth, and that the expression of IKKα is positively linked with the survival of NPC patients and IKKα may be used as a novel target for differentiation therapy in NPC [15–18]. These findings demonstrated that IKKα is diversely expressed in keratinizing and non-keratinizing NPC.

**Figure 2:**
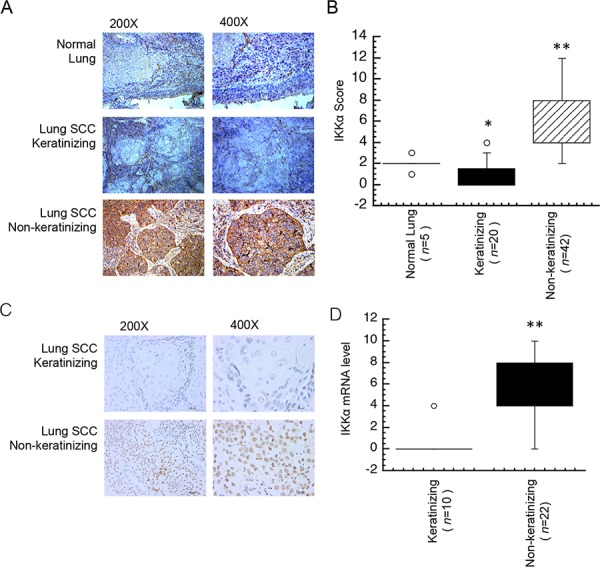
Different expression level of IKKα in lung SCC tissues **A.** Immunohistochemical analysis was used to examine the protein level of IKKα in a normal lung and tissues from NPC patients. **B.** Expression protein level of IKKα in lung and subtypes of lung SCC tissues as indicated. **C.** The mRNA level of IKKα was analyzed by ISH in lung cancer tissues as indicated. **D.** Expression mRNA level of IKKα in subtypes of lung caner tissues as indicated. *n*, number of analyzed samples, * *p* < 0.05, ** *p* < 0.01.

Lung cancer is generally classified into a number of types including small cell carcinoma, squamous cell carcinoma (SCC), adenocarcinoma (ADC) and large cell carcinoma. SCC has been classified only by the degree of keratinization as a parameter of differentiation [19]. To further address the role of IKKα in keratinizing and non-keratinizing cancer, we chose five cases of normal lung, 20 case of SCC with keratinization and 42 cases of SCC with non-keratinization for this study. As expected in Figure 2A, weak IKKα staining was displayed in keratinizing SCC and strong IKKα staining was displayed in non-keratinizing SCC samples, moreover, the score of IKKα expression in keratinizing lung SCC was significantly lower as compared with non-keratinizing lung and non-cancer tissues (Figure 2B). Next, we used *in situ* hybridization (ISH) to analyze the mRNA level of IKKα in lung cancer. A total of 32 keratinzing and non-keratinizing lung cancer samples were evaluated with ISH. Data in Figure 2C showed that IKKα was significantly absence in non-keratinizing lung cancer, while IKKα increased in non-keratinizing lung SCC. Moreover, the score of IKKα mRNA expression in keratinizing lung SCC was significantly lower as compared with non-keratinizing lung SCC (Figure 1D). It indicated that loss of IKKα showed in keratinizing lung SCC and IKKα gained in non-keratinizing lung SCC.

**Figure 3:**
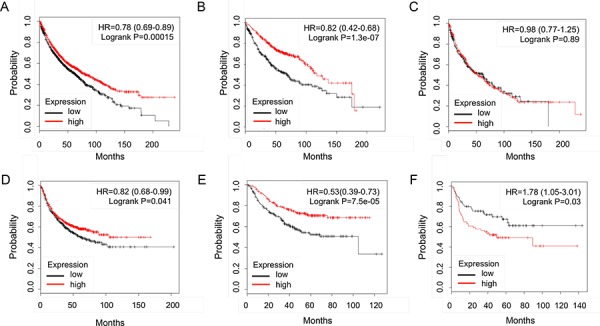
An in silico meta-analysis of IKKα expression profiles with Kaplan-Meier plotter in lung cancers Overall survival analyzed in all lung cancer **A.** adenocarcinoma **B.** and SCC **C.** Fist progression analyzed in all lung cancer **D.** adenocarcinoma **E.** and SCC **F.**

To further address the role of IKKα in lung cancers, an *in silico* meta-analysis of IKKα expression profiles with Kaplan-Meier plotter (https://kmplot.com) performed on a cohort of lung cancers showed that higher expression of IKKα gene linked with overall survival in all lung cancer (1926 cases, Figure 3A) and adenocarcinoma (719 cases, Figure 3B), but not in lung SCC (525 cases, Figure 3C). Moreover, the higher expression of IKKα gene was correlated with first progression in all lung cancer (982 cases, Figure 3D) and adenocarcinoma (461 cases, Figure 3E). However, the higher expression of IKKα reversely was correlated with first progression in human lung SCC (141 cases, Figure 3F). It hints that IKKα may just function as initiator for cancer progression in lung SCC while IKKα only functions as a tumor suppressor in lung adenocarcinoma. Clearly, the analysis is contrast to the findings that IKKα reduction is associated with the development of spontaneous lung SCC in mice, which are associated with IKKα downregulation and inflammation [12].

Interestingly, IKKα comprises a nuclear localization signal and therefore also confers important nuclear functions. In the nucleus, IKKα forms an intact complex with CREB binding protein (CBP) and contributes to NFκB promoted gene expression through phosphorylation of histone H3 [20–22]. Additional tumor-promoting nuclear functions of IKKα include cell cycle regulation and chromosomal accessibility by phosphorylation of histone H3, Aurora B kinase, or the nuclear corepressor SMRT, which triggers its nuclear export with HDAC3 and its degradation [23, 24]. Phenotypic plasticity and functional heterogeneity are important features of tumors arising in various organs [25, 26]. Interestingly, depletion of Liver kinase B1 (Lkb1), a tumor suppressor encodes an evolutionarily conserved serine/threonine kinase, may initiate the transdifferentiation of lung adenocarcinoma in mice to SCC [27]. Whether IKKα shows the similar roles in the transdifferentiation remains further identification.

The epidermis is a squamous epithelium where keratinocytes follow a unique programme of terminal differentiation and cell death that leads to the formation of the cornified layer, the outermost skin barrier [28]. Keratinization is observed in the skin, tongue and external half of the lips where keratins produce the formation of keratinization and cornification in skin modifications [29]. The somatic conditional depletion of IKKα using keratin 5 or keratin 14 promoters leads to tumor progression [12, 30]. However, in skin carcinogenesis assays, transgenic mice carrying active Ha-ras (K5-IKKα-Tg.AC mice) also develop invasive tumors, instead of the benign papillomas arising in wild type-Tg-AC mice also bearing an active Ha-ras [31], indicating a tumor promoter role of IKKα in skin cancer, similarly to what occurs in other neoplasias, including hepatocarcinomas [32], breast [33], prostate [34] and colorectal cancer [35]. Keratinzed or non-keratinized epithelial is based historically on the notion that only the epidermis of skin modifications such as horns, claws and hooves is cornified, that non-modified epidermis is a keratinized stratified epithelium, and that all other stratified and non-stratified epithelia are non-keratinized epithelia [29]. Several factors such as keratins, filaggrin, loricrin, transglutaminases play critical roles in keratinzation and keratinizing disorders in keratinocytes [36]. Recently, TMEM45A gene belongs to the large family of gene coding uncharacterized predicted transmembrane (TMEM) proteins and demonstrates that TMEM45A is strongly linked with epidermal keratinization [37]. It is not sure whether these factors contribute epidermal differentiation is a multi-step process regulated by specific pro-differentiation. The role of these factors in transdiffernetion of keratinizing and non-keratinzing SCCs from epidermis remains poorly known. Moreover, it is unclear that which factors and how initiate the transition between keratinizing and non-keratinizing cancer from epithelial cells. Whether and how IKKα as well as nuclear IKKα involves in the transition of NPC between keratinizing and non-keratinizing remains further identification.

Taken together, we found that IKKα expression was not expressed in keratinizing cancer but in non-keratinizing cancer, and that IKKα expression was linked with cellular differentiation in non-keratinizing NPC. These findings hint that IKKα is diversely expressed in keratinizing and non-keratinizing carcinomas in the same type of cancer.

## MATERIALS AND METHODS

### Tissue samples and clinical information

The ethical committee of our hospital approved the study. Archival materials with a diagnosis of keratinizing and non-keratinizing nasopharyngeal carcinoma, keratinizing and non-keratinizing lung squamous cell carcinoma were retrieved from the files of Xiangya Pathologic Anatomy Service. A total of seventy cases (5 of normal nasopharynx, 43 of NPC, 5 normal lung, and 62 of lung SCC) fit criteria for inclusion study. The histologic sections of all cases were re-reviewed and the diagnoses confirmed by the pathologist. Clinical information was extracted from the medical records. All patient data were de-identified.

### Immunohistochemical analysis and ISH of biopsies

Five-micrometer-thick sections were obtained for immunohistochemical studies, which were performed on formalin-fixed, paraffin-embedded tissues using standard perosidase immunohistochemistry techniques, heat-induced epitrope retrieval butffer and primary antibodies against IKKα (Cat # IHC-00401, Bethyl). Appropriate positive and negative controls were included. All stained slides were initially reviewed and scored by the first author and re-viewed by three pathologists in a blinded fashion to ensure consistency of interpretation.

ISH was performed using the biotin labeled probe from ISH kit (Life technologies), according to the instruction of the manufacturers. The probe sequence was following: 5′-CAATGTGTTCTAGATGGAGTTAGAGGCTGTG ATAGCTATATGGTT-3′.

IKKα staining was considered positively by ascertaining cytoplasmic and nuclear expression. The determination result was obtained from semi-quantitative classification according to 10 more visual fields (×200). The slides were first scored as 0 (negative), 1 (buff), 2 (pale brown), and 3 (tan). Positive expression of IKKα were scored as 0 (negative), 1+ (<10% of positively-staining tumor cells), 2+ (11–50% of positively-staining tumor cells), 3+ (50–75% of positively-staining tumor cells), and 4+ (>75% of positively-staining tumor cells. Both the scores by multiply were regarded as the determination result.

#### Statistical analysis and an *in silico* meta-analysis

The statistical association of expression of IKKα was analyzed using the SPSS 10.0 software. A two-tailed *P* value of less than 0.05 and 0.01 was considered to be statistically significant and much significant respectively. An *in silico* meta-analysis of IKKα expression profiles with Kaplan-Meier plotter from the webstie (https://kmplot.com).
